# Relationship Status and Relationship Instability, but Not Dominance, Predict Individual Differences in Baseline Cortisol Levels

**DOI:** 10.1371/journal.pone.0084003

**Published:** 2013-12-16

**Authors:** Dario Maestripieri, Amanda C. E. Klimczuk, Marianne Seneczko, Daniel M. Traficonte, M. Claire Wilson

**Affiliations:** Institute for Mind and Biology, The University of Chicago, Chicago, Illinois, United States of America; University of Lausanne, Switzerland

## Abstract

We investigated variation in baseline cortisol levels in relation to relationship status (single or in a relationship), relationship characteristics (length, stability, presence or absence of clear dominance), or individual attributes (dominant or subordinate status, relative physical attractiveness, relationship worries). Study participants were 77 men and 75 women aged between 18 and 38 years. Individuals in romantic relationships had lower cortisol levels than singles. Individuals of African ethnicity, however, showed the opposite pattern. Individuals who perceived their relationship to be highly unstable had higher cortisol levels. Aside from African-Americans, married individuals reported the lowest relationship instability and the lowest cortisol levels, followed by individuals in long-term relationships, and by individuals in short-term relationships. The presence or absence of clear dominance in the relationship, dominance status, or relationship worries did not affect cortisol levels. Therefore relationship status and relationship instability were better predictors of variation in cortisol (presumably through stress-related mechanisms) than individual attributes.

## Introduction

In the study of social relationships, as in many other areas of psychology, there has been increasing interest in understanding the bidirectional relationships between behavior and the body. In this regard, a growing number of human and animal studies have shown that hormones play an important role in the establishment, maintenance, and overall quality of social relationships, while endocrine function in turn is affected by the formation or disruption of relationships or by their quality [1,2]. 

Stress hormones, particularly cortisol, have figured prominently in research on the endocrinology of human relationships. A number of studies highlighting the benefits of social relationships for mental and physical health have shown that lonely people have higher cortisol levels when compared to people who are embedded in supportive social networks [3,4]. Another body of work has shown that individuals with secure attachment to their romantic partner exhibit lower cortisol responses to stress [5,6]. Relatively little research has been conducted on the association between relationship functioning and interindividual variation in cortisol (but see 7). For example, little is known about whether individuals who are in stable vs unstable relationships exhibit different cortisol profiles [8]. Understanding the association between relationship functioning and cortisol, however, is important from a theoretical perspective because stress hormones play an important role in mediating the influence of the social environment on life history strategies and because variation in relationship functioning is an important factor that may differentiate slow and fast life histories [9,10]. From a life-history perspective, cortisol levels may not only reflect stress but also be functionally linked to life-history trade-offs such as those between self-maintenance activities and reproduction (for these effects of corticosterone in animals, see 11). 

Evidence from nonhuman primates indicates that individuals in unstable relationships have higher cortisol levels than those in stable relationships [12,13]. In nonhuman primates, dyadic social relationships (between two males, two females, or a male and a female) have a strong dominance component, such that one member of the pair is dominant and the other is subordinate. One major source of relationship instability is uncertainty and fighting over dominance. Such relationship instability clearly entails stress. Cortisol is a metabolic hormone that mobilizes energy at times when such energy is needed. Therefore the increase in cortisol observed in these individuals with unstable relationships is consistent with the key function this hormone plays in priming the body for dealing with challenges. Aside from relationship instability, dominance status in itself can be associated with stress and with high or low cortisol levels depending on the circumstances [14]. 

Human romantic relationships have a dominance component, too, such that one partner is typically dominant and the other is subordinate [15]. Unfortunately, no previous research has investigated cortisol profiles in couples in relation to the presence or absence of dominance, and each partner’s dominant or subordinate role within the relationship. Whether romantic relationships are good or poor, stable or unstable, or with or without dominance may depend on many different factors including the individuals’ physical attractiveness and personality characteristics; their attachment styles, social and cultural norms concerning romantic relationships; and their health, socioeconomic status and other stressors or supporting factors present in the environment. Some of these factors are also known to affect cortisol profiles (e.g., for effects of ethnicity and socioeconomic status on cortisol, see 16,17. 

The purpose of this study was to investigate interindividual variation in cortisol levels in relation to relationship status (i.e., whether individuals are single or in a romantic relationship), relationship characteristics (e.g., length, stability, and the presence of clear dominance), and the individual’s dominance status (dominant or subordinate) within the relationship. Our study participants were not tested in stressful conditions, so we interpret their cortisol concentrations as their baseline profiles and assume that they reflect the general degree of stress experienced by the study participants in their lives at the time the study was conducted.

Our first hypothesis was that being in a romantic relationship is generally beneficial for psychological well-being and associated with a lower level of stress than being single; we therefore tested the prediction that individuals in relationships should have lower cortisol levels than single individuals. To investigate whether this pattern generalizes across ethnic groups that may differ in cultural ideals, socioeconomic status, or behavior in relationships, we compared four ethnic groups: Caucasians, Africans, Hispanics, and Asians. Ethnic differences in the association between cortisol and relationship status might also emerge as a result of ethnicity-related variation in relationship stability. We therefore tested the hypothesis that relationship instability would be associated with higher cortisol levels both at the individual level and at the level of ethnic group. 

We then investigated some possible predictors of relationship instability and of variation in cortisol at the relationship level and at the level of the individual. We hypothesized that long-term relationships, and especially those involving marriage, should be more stable than short-term relationships and also be associated with lower levels of cortisol. We assessed whether relationship stability/instability and cortisol levels are affected by the presence of clear or unclear dominance in the relationship. Here we tested two competing hypotheses with opposite predictions. The first hypothesis (H1) is that relationships with clear dominance (non-egalitarian) are highly stable and relatively non-stressful (because dominance is accepted and undisputed by both parties) when compared with relationships without clear dominance (egalitarian); this hypothesis predicts that cortisol levels should be lower in individuals in non-egalitarian vs egalitarian relationships. The second hypothesis (H2) is that relationships with clear dominance are less stable and more stressful (because dominance is disputed) than egalitarian relationships; this hypothesis predicts that cortisol levels should be higher in individuals in non-egalitarian vs egalitarian relationships. At the individual level, we tested whether individuals who are dominant or subordinate in their relationships have relatively high or low cortisol levels. We also investigated the extent to which being dominant or subordinate in a relationship predicts the level of worry about the relationship and whether, in turn, relationship worries predict individual cortisol levels.

## Methods

### Study participants

Study participants were 77 men and 75 women aged between 18 and 38 years (mean + SE= 22.72 + 0.32 years). All study participants completed a written informed consent form before participating in the study and were paid $ 20 after completion of the procedures. This study and the use of human subjects were approved by the Social Sciences Institutional Review Board of the University of Chicago. Approximately 80% of the study participants were undergraduate or graduate students at the University of Chicago, while most of the others were employed by the same university under various capacities. They were recruited on the University of Chicago campus through fliers, mailing lists, or a human subject recruitment website (Sona System). 

### Procedure

An initial survey asked information about participants’ age, ethnicity, sexual orientation, and relationship status (single or in a relationship; for some data analyses, the relationship category was divided into three subgroups: short-term relationship, if less than 6 months; long-term relationship, if more than 6 months; and marriage). Of the 152 study participants, 78 of them were Caucasian, 23 were Hispanic, 28 were Asian, and 23 were African. 133 participants self-described their sexual orientation as heterosexual, 9 as homosexual, and 10 as bisexual. 55 study participants were single and 97 were in a relationship. The participants included 19 heterosexual and two homosexual couples.

Other questionnaires asked questions about personality characteristics and romantic relationships. For the purposes of this study, the following four questionnaire items were considered: 

#### Relationship instability

Study participants were asked: “Think about your current romantic relationship. Would you describe it as stable (little tension, little uncertainty, few break-ups) or unstable (a lot of tension, a lot of uncertainty, many break-ups)?” Answers were given on 1-7 scale, with 1= very stable, and 7= very unstable.

#### Dominance

Study participants were asked: “In your current romantic relationship, which of you is more dominant?” Answers were given on 1-5 scale, with 1= I am definitely dominant to my partner, 2= I am somewhat dominant; 3= neither one of us is dominant; 4= my partner is somewhat dominant, and 5= my partner is definitely dominant. If a score of 1 or 2 was used, the participant was classified as dominant; if a score of 4 or 5 was used, the participant was classified as subordinate; if a score of 3 was used, dominance status was not assigned. Participants who gave a 3 score were considered to be in egalitarian relationships. Participants who gave any score other than 3 were considered to be in non-egalitarian relationships.

#### Partner attractiveness

Study participants were asked: “In your current romantic relationship, who attracts more sexual/romantic attention from other men/women?” Answers were given on 1-5 scale, with 1= I definitely attract a lot more attention, and 5= my partner definitely attracts a lot more attention.

#### Worries about the relationship

Study participants were asked: “In your current romantic relationship, who is more worried about the relationship ending?” Answers were given on 1-5 scale, with 1= I am definitely more worried, and 5= my partner is definitely more worried.

Single individuals were asked to answer the four questions with reference to their most recent romantic relationship. However, relationship data from single individuals were not included in the data analyses of this study.

At the beginning of the procedures, participants were asked to provide a saliva sample. All saliva samples were collected between 1pm and 4pm. Previous studies have shown that although cortisol concentrations are lower in the afternoon than in the morning, afternoon hormone levels are more stable and therefore better suited for studies of social endocrinology (e.g. [18]; indeed, there was no significant effect of time of collection in relation to time of day on cortisol levels in this study; R^2^= 0.006; F _1,121_= 0.71; p = 0.40). Saliva was collected into a plastic tube. Saliva samples were stored in ice and later shipped to the University of New Mexico, where they were assayed for cortisol using ELISA reagents. The cortisol antibody (R4866) cross-reacts with cortisone (5%), but all other cross-reactivities with endogenous steroids are < 1%. Sensitivity of the assay is approximately 16 pg/ml. Inter-assay CV was 12% for low sample and 9% for high sample. Intra-assay CV (mean CV of duplicates) was 4%. Cortisol data were available for a subset of study participants (n= 122; 62 males and 60 females).

## Results

### Relationship status, ethnicity, and cortisol levels

A multiple regression analysis indicated that variation in cortisol levels was not significantly predicted by variation in age, gender (mean cortisol + SE; male= 4229.26 + 184.32; female= 4686.08 + 259.32) or ethnic group (mean cortisol + SE; Caucasian= 4322.37 + 197.69; Hispanic= 5064.68 + 413.68; Asian= 4287.52 + 353.38; African= 4559.24 + 518.54) (R^2^= 0.03; F _3,120_= 1.38; p = 0.19). 

**Figure 1:**
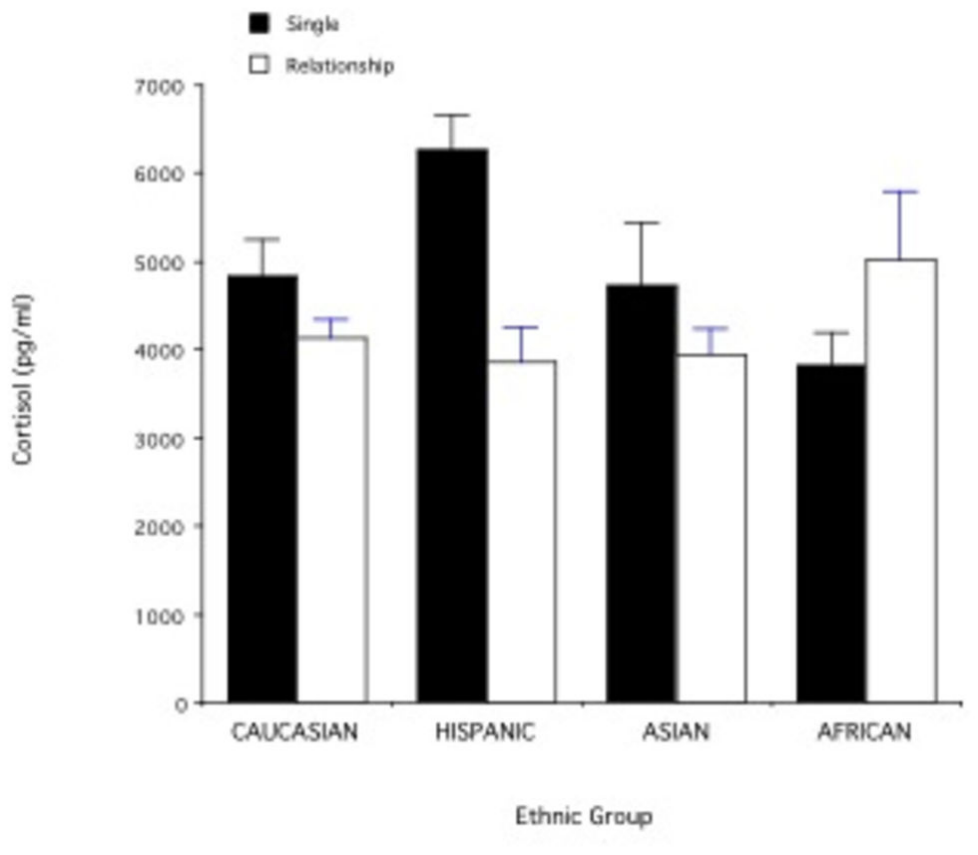
Cortisol levels in relation to ethnic group and relationship status. Sample sizes: Caucasians (single= 17; relationship= 45), Hispanics (single=8; relationship= 8), Asians (single=10; relationship= 13), Africans (single=8; relationship= 13).

Individuals who were in relationships (n= 79) had significantly lower cortisol levels (mean + SE= 4220.96 + 190.74) than singles (n= 43; mean + SE= 4881.93 + 274.98; t= -2.01; df= 120; p< 0.05). When ethnicity, however, was taken into account, the effect of relationship status on cortisol failed to reach significance (F _1,114_= 3.54; p= 0.06) but there was a significant interaction between ethnicity and relationship status (2 x 2 ANOVA: F _3,114_= 3.41; p= 0.02). Figure 1 illustrates that while for Caucasians, Hispanics, and Asians single individuals had higher average cortisol than individuals in relationships, the opposite was true for Africans (p < 0.05). 

**Figure 2:**
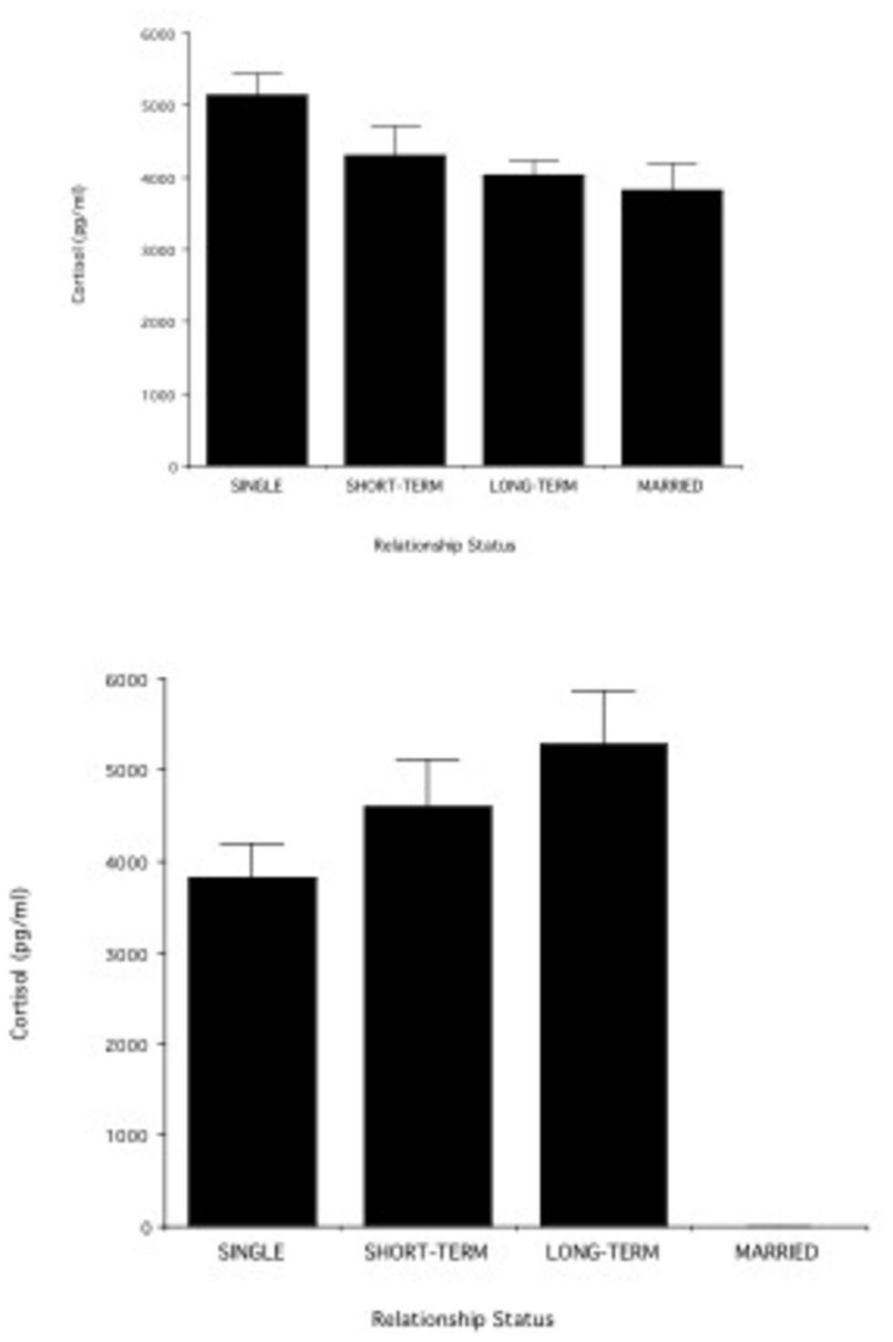
(a). Cortisol levels in individuals who are single (n= 35), in short-term relationships (n= 17), in long-term relationships (n= 38), or married (n= 11). Data are pooled for Caucasians, Hispanics, and Asians. (b) Cortisol levels in African individuals who are single (n= 8), in short-term relationships (n= 5), or in long-term relationships (n= 9).

When data for Caucasians, Hispanics, and Asians were pooled together and relationship status was divided into three subgroups (short-term, long-term, and married), single individuals had the highest cortisol levels, individuals in short-term relationships had the next highest cortisol levels, followed by individuals in long-term relationships and by married individuals, who had the lowest cortisol levels (Figure 2a; F _3,100_= 3.80; p= 0.01). Bonferroni-Dunn post-hoc tests indicated that singles were significantly different from individuals in long-term relationships and married (p < 0.05); other individual group comparisons were not statistically significant. Figure 2b shows similar data for Africans. In this ethnic group there were no married individuals. Cortisol levels were, on average, highest in the long-term relationship subgroup, followed by the short-term relationships, followed by the singles, who had the lowest cortisol levels. The difference among the three groups, however, was not significant (F _2,20_= 0.73; p = 0.49).

### Relationship instability, ethnicity, and cortisol

**Figure 3:**
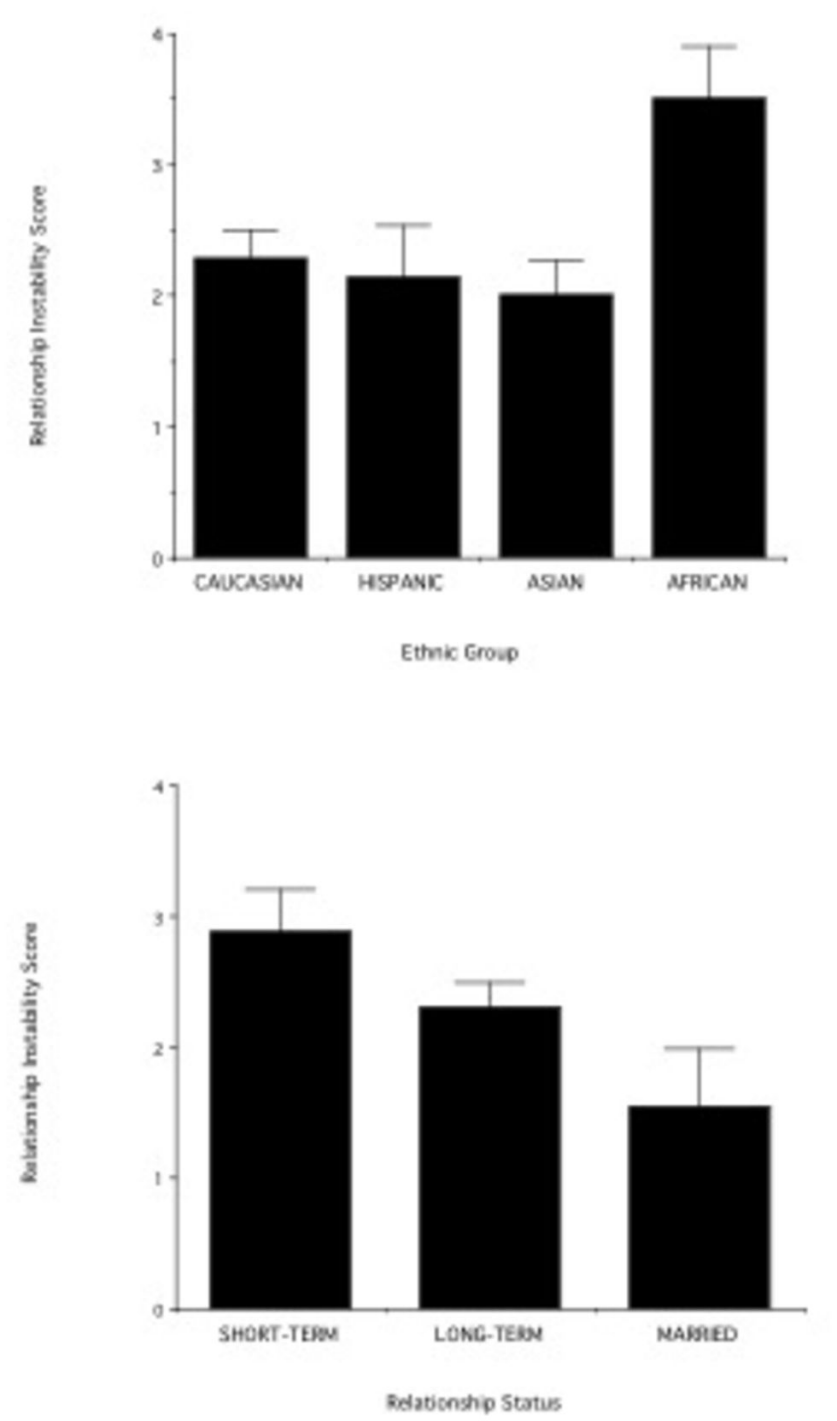
(a) Relationship instability in relation to ethnic status. The higher the score the more unstable the relationships. (b) Relationship instability among individuals who are in short-term relationships, in long-term relationship, or married. Data from all four ethnic groups are combined.

Among study participants who were in relationships, perceived relationship instability was significantly positively correlated with cortisol levels (log-transformed data) across genders and ethnic groups (r= 0.27; n= 79; p= 0.015). Thus, individuals who perceived to be in more unstable relationships had higher cortisol levels. Relationship instability was also significantly different across ethnic groups (F _3,98_= 2.88; p= 0.02). Figure 3a shows that Africans had more unstable relationships than the other ethnic groups (post-hoc tests, all p< 0.05). 

There was a significant difference in perceived relationship instability among married individuals and unmarried individuals in long-term and short-term relationships (data from all four ethnic groups were combined for this analysis) (F _2,96_= 3.07; p= 0.05; Figure 3b). Individuals in short-term relationships reported significantly higher instability scores than married individuals (post-hoc test, p < 0.05). Individuals in long-term relationships reported instability scores that were intermediate between (but not significantly different from) those of individuals in short-term relationships and those of married individuals. In the African ethnic group, short-term relationships were more unstable than long-term ones (short-term= 5.0 + 0.55; n= 5; long-term= 2.67 + 0.44; n= 9; t= 3.24; df= 12; p= 0.007).

There were no significant differences between individuals in egalitarian (n= 23) and in non-egalitarian relationships (n= 55) in cortisol levels (mean + SE, egalitarian= 4196.96 + 481.05; non-egalitarian= 4257.36 + 187.93; t= 0.14; df= 76; p = 0.88) or in relationship instability (mean + SE, egalitarian= 2.13 + 0.34; non-egalitarian= 2.24 + 0.17; t= 0.31, df= 76; p = 0.75). 

### Dominance, relationship worries, and cortisol levels

**Figure 4:**
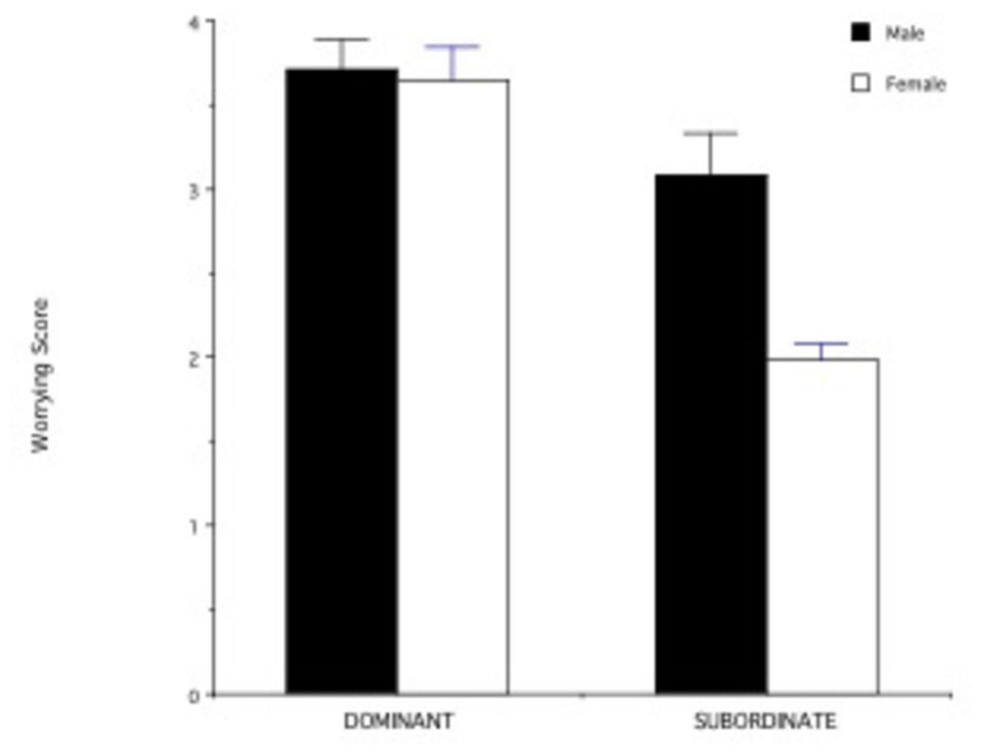
Scores for the “Worrying about the relationship” scale in relation to dominance status within the relationship and gender. A low score indicates that the individual is more worried about the relationship than his/her partner; a high score indicates that the individuals thinks that his/her partner is more worried than himself/herself.

For heterosexual participants in non-egalitarian relationships, males who were dominant in the relationship (n=24) were more than twice as many as males who were subordinate (n=11), whereas there were slightly fewer dominant (n=11) than subordinate females (n=13) (chi square= 3.05; df=1; p= 0.08). Dominant or subordinate status within a relationship was not significantly associated with perceived relative attractiveness (dominant = 2.77 + 0.18, n= 35; subordinate = 2.80 + 0.26, n= 25; t= -0.09; df= 58, p = 0.93). For all participants (both heterosexual and homosexual) in non-egalitarian relationships, subordinates worried disproportionately more than dominants about a possible relationship break-up (F _1, 61_= 13.78; p < 0.001); females, however, worried more than males regardless of their dominance status (F _1,61_= 4.02; p < 0.05; Figure 4). This effect was mainly driven by subordinate females, who worried more than any other group (post-hoc tests: p< 0.05; see Figure 4). 

The possible association between dominance and cortisol was assessed in two different ways. First, we compared cortisol levels between individuals who reported being dominant or subordinate in their relationships (individuals in egalitarian relationships were not included). There was no significant difference in cortisol between dominants and subordinates (dominants= 4348.51 + 226.96; n= 33; subordinates= 4120.63 + 328.37; n= 22; t= 0.59; df= 53; p = 0.55). Second, we assessed a correlation between dominance scores (see Methods) and cortisol levels. This analysis included also individuals with a 3 dominance score, who were in egalitarian relationships. There was no significant correlation between cortisol levels and dominance scores (n=78; r= 0.08; p = 0.57). Finally, there was also no significant correlation between relationship worries and cortisol levels (n= 78; r= 0.04; p = 0.68). 

## Discussion

In a subject population consisting mainly of university students, relationship status was a significant predictor of cortisol levels, as men and women in romantic relationships had significantly lower cortisol levels than men and women who were single. This effect, however, was driven by individuals of Caucasian, Hispanic, and Asian ethnicity, who altogether comprised approximately 83% of the study participants for whom cortisol data were available. Individuals of African ethnicity showed an opposite effect of relationship status on cortisol levels, such that men and women in relationships had higher cortisol levels than men and women who were single. The sample size for individuals of African ethnicity, however, was small (n=21). Therefore, the observed difference in cortisol levels between singles (n= 8) and individuals in relationships (n= 14) in this ethnic group needs to be replicated in a larger sample.

 A possible explanation for our findings is that for most individuals the single lifestyle is associated with more stress than the romantic relationship lifestyle (see also 4,19. Being in a relationship, however, is associated with low cortisol (and therefore, presumably low stress) only if the relationship is stable. Among our study participants, relationship instability was a predictor of variation in cortisol levels across genders and ethnic groups: individuals in highly unstable relationships had higher cortisol levels. Individuals of African ethnicity had more unstable relationships than individuals in other ethnic groups and this may have contributed to the different association between relationship status and cortisol observed in this group. The relationship instability in the African ethnic group may have been due to socioeconomic or cultural variables. No firm conclusions can be drawn, however, about ethnicity and relationship instability until our results are replicated with a larger sample size.

 Across all ethnic groups, short-term relationships were more unstable than long-term relationships, which in turn were more unstable than marriages. Among non-Africans, variation in cortisol levels tracked the variation in instability among relationship types: cortisol was highest among individuals in short-term relationships, followed by individuals in long-term relationships, followed by married individuals. Among participants of African ethnicity, cortisol levels showed an opposite pattern of variation: cortisol levels were, on average, highest in the long-term relationship subgroup, followed by the short-term relationships, followed by the singles, who had the lowest cortisol levels.

The causes of relationship instability can be multiple and heterogenous. We found no significant differences in perceived relationship instability or in cortisol levels between individuals in egalitarian vs non-egalitarian relationships. In non-egalitarian relationships, dominant or subordinate status was not clearly associated with gender (but more males than females tended to have a dominant role) or with relative physical attractiveness (i.e., the partner that was perceived to be more attractive and to receive more sexual or romantic attention from others was not necessarily dominant or subordinate in the couple). Subordinates worried disproportionately more than dominants about a possible relationship break-up, and females worried more than males regardless of their dominance status. The hypothesis that being subordinate and worrying more about the relationship is associated with higher cortisol level, however, was not supported by our data. 

Thus, the characteristics of the relationship as a whole, particularly its perceived instability, were a better predictor of variation in cortisol levels than the individual’s attributes: whether the individual was more or less attractive than his or her partner, whether the individual was dominant or subordinate, and whether the individual worried more or less than his or her partner about the future of the relationship. It is possible that dominance has stronger effects on stress and cortisol in couples of older adults, who have lived together for longer periods of time, and in which the dynamics of interaction between partners are well established and highly consistent over time. The main source of stress in relationships between young adults, such as the participants in this study, may not be dominance-related asymmetries in the relationships, but uncertainty over whether or not the relationship will last. It is remarkable, however, that even among young adults, whose lives are still mainly focused on education and their future careers, social variables are significant predictors of stress-related physiological variables. 

Further research is needed to better understand how individual characteristics (e.g. personality traits, adult attachment styles, or early family experiences) may influence the perception of relationship stability/instability and how one’s perception of relationship characteristics or one’s own role (e.g. dominant or subordinate) within the relationship may influence specific aspects of stress-sensitive physiological systems such as the hypothalamic-pituitary-adrenal (HPA) axis. The assessment of HPA activity through the measurement of cortisol concentrations in a single afternoon saliva sample is one of the methodological limitations of this study. Future studies examining the effects of social relationships on HPA activity should include multiple saliva samples collected at different times of day (to examine such issues as the cortisol awakening response and its relation to sleep pattern, circadian variation in cortisol levels, and total hormone production under the curve) as well as include samples collected both in “baseline” standardized conditions and in response to challenges (e.g. a laboratory test of psychosocial stress) [20,21]. It is also important that these future studies be conducted with subject populations other than those consisting mainly of college students to make sure that the findings are generalizable. Given that variation in cortisol levels is known to influence many aspects of behavior, emotion, and cognition [1,2], another promising venue for future research would be the investigation of how variation in HPA activity resulting from social variables in turn affects our preferences, our decisions, and more generally our behavior in both social and non-social contexts, including contexts that important to survival and reproductive success such as nutrition, mating and reproduction, and investment in children. 
